# Genetics of Hypertriglyceridemia

**DOI:** 10.3389/fendo.2020.00455

**Published:** 2020-07-24

**Authors:** Jacqueline S. Dron, Robert A. Hegele

**Affiliations:** Departments of Medicine and Biochemistry, Schulich School of Medicine and Dentistry, Robarts Research Institute, Western University, London, ON, Canada

**Keywords:** autosomal recessive, complex trait, familial chylomicronemia syndrome (FCS), multifactoriel chylomicronemia (MCM), polygenic score, triglyceride

## Abstract

Hypertriglyceridemia, a commonly encountered phenotype in cardiovascular and metabolic clinics, is surprisingly complex. A range of genetic variants, from single-nucleotide variants to large-scale copy number variants, can lead to either the severe or mild-to-moderate forms of the disease. At the genetic level, severely elevated triglyceride levels resulting from familial chylomicronemia syndrome (FCS) are caused by homozygous or biallelic loss-of-function variants in *LPL, APOC2, APOA5, LMF1*, and *GPIHBP1* genes. In contrast, susceptibility to multifactorial chylomicronemia (MCM), which has an estimated prevalence of ~1 in 600 and is at least 50–100-times more common than FCS, results from two different types of genetic variants: (1) rare heterozygous variants (minor allele frequency <1%) with variable penetrance in the five causal genes for FCS; and (2) common variants (minor allele frequency >5%) whose individually small phenotypic effects are quantified using a polygenic score. There is indirect evidence of similar complex genetic predisposition in other clinical phenotypes that have a component of hypertriglyceridemia, such as combined hyperlipidemia and dysbetalipoproteinemia. Future considerations include: (1) evaluation of whether the specific type of genetic predisposition to hypertriglyceridemia affects medical decisions or long-term outcomes; and (2) searching for other genetic contributors, including the role of genome-wide polygenic scores, novel genes, non-linear gene-gene or gene-environment interactions, and non-genomic mechanisms including epigenetics and mitochondrial DNA.

## Introduction

Circulating triglyceride (TG) levels above 2.0 mmol/L (175 mg/dL) is defined as “hypertriglyceridemia,” and is commonly encountered in cardiovascular and metabolic clinics. Technological advances in DNA analysis have allowed for the molecular genetics of hypertriglyceridemia to be explained in a large proportion of patients. At the genetic level, hypertriglyceridemia is usually complex in nature; only the very rare autosomal recessive familial chylomicronemia syndrome (FCS) phenotype shows Mendelian inheritance. There is no highly penetrant form of autosomal dominant hypertriglyceridemia that would be analogous to familial hypercholesterolemia (FH). Instead, there are multiple types of genetic contributors to hypertriglyceridemia: both rare and common DNA variants. These variants create a state of susceptibility to hypertriglyceridemia but are not absolutely causative. In this review, we discuss the types of genetic variation underlying hypertriglyceridemia and the different underlying genetic effects that are determinants of clinical expression of this condition.

## Overview of Genetic Variation

### Types of Genetic Variants

We briefly review the types of human genetic variation that contribute either causally or indirectly to hypertriglyceridemia. The term “variant” is favored over “mutation,” since the former is more versatile, while the latter may have negative connotations; genetic variants can have either damaging or beneficial effects. The term “variant” carries no implications related either to its frequency in the population or its potential dysfunction and pathogenicity. Particular biological properties are specified by adjectives applied to the term “variant” (1).

Most often, genetic variation results from the existence of two (or more) differences across a particular genomic locus; these differences are defined as “variants” (2). When these differences occur in a gene, the encoded protein's normal function may be disrupted. These alternative gene possibilities are called “alleles.” A rare variant is conventionally defined as having an allele frequency <1% in the population, while a common variant has an allele frequency >5% (1). Variants with frequencies between 1 and 5% are referred to as uncommon variants. Broadly, a genetic variant's frequency in the population can be inversely correlated with its pathogenicity, e.g., variants that alter the protein-coding sequence of an important gene are more likely to exert a phenotypic or pathogenic effect and tend to be selected against, resulting in their rarity in the population. However, there are countless examples of phenotypically neutral rare variants and some examples of common variants with moderate effects, such as the apolipoprotein (apo) E E4/E3/E2 isoforms.

The types of human genetic variation can be broken down into two main categories: (1) single-nucleotide variants (SNVs), which are qualitative changes in the DNA sequence that involve a single nucleotide—a familiar subtype of SNV is the common single-nucleotide polymorphism (SNP); and (2) all other changes, which are larger than a single nucleotide base (3). The latter group includes structural variations such as insertions, and deletions and duplications of entire portions of a gene or chromosome; such structural variations are collectively called “copy-number variants” (CNVs), whose consequences include alteration of the dosage or copy-number of nucleotides within a particular genomic region (3).

The vast majority of genomic SNVs mostly occur within non-coding intergenic or intronic regions (2). SNVs within coding regions are referred to as “synonymous” if they do not directly affect the amino acid sequence or “nonsynonymous” if the amino acid sequence is altered. A SNV can introduce missense, nonsense, and stop-gain variants, or it may affect RNA splicing by altering sequences at intronic splice donor or acceptor sites, or by activating cryptic splice enhancers within coding sequences (3).

The second variant type, CNVs, are quite abundant in the genome (3). CNVs can range from small insertions and deletions (indels) spanning 1–50 base pairs, to small deletions or duplications (del-dups) of genetic material, through to very large cytogenetic changes that involve entire chromosomes (e.g., trisomy 21). A CNV deletion involving part of or an entire gene often cripples transcription and translation, although the size of the CNV does not always correlate with dysfunctional outcomes. Smaller CNVs can also affect the coding and translation of a protein. In contrast, a germline duplication of a whole gene may result in higher expression of the gene product with clinical consequences (4).

### Functional Impact of Genetic Variants

Genetic variants determine clinical phenotypes, like hypertriglyceridemia, when the DNA sequence change results in a structural or functional consequence affecting the protein product within a key metabolic or biochemical pathway. Qualitatively, the DNA alteration can lead to a loss or gain in protein function or have a neutral functional impact. For a loss-of-function (LOF) variant, the protein product's function is diminished or lost either through decreased expression or a compromising structural change. In contrast, a gain-of-function (GOF) variant enhances the normal function of the protein product, either through increased expression or change in protein structure that increases its functional activity or efficiency. A further dimension of the functional changes resulting from a genetic variant is quantified by the magnitude of the altered function compared to baseline wild-type function. Most genetic variants that affect TG metabolism are LOF, but a few display a gain in function, such as the rare *APOC3* p.Gln38Lys variant that raises TG levels (5) and the common *LPL* p.Ser474Ter (a.k.a. p.Ser447Ter) variant that lowers TG levels (6). Interestingly, variants disrupting microRNA binding sites in the 3′-untranslated region of *LPL* are reported to be in linkage disequilibrium with p.Ser474Ter, which may suggest that the disruption in gene regulation may also contribute to the GOF mechanism (7, 8).

### Analyzing Genetic Variants

The classification of the genetic determinants of hypertriglyceridemia guides the development of methodologies to detect them (9, 10). Because both common and rare variants, including SNVs and CNVs, are involved in the pathogenesis of hypertriglyceridemia, an optimal diagnostic method must be able to detect all possible variant types and bioinformatically process them en route to reporting them for potential clinical or research applications. In our experience, this is best accomplished using a targeted next-generation sequencing panel for hypertriglyceridemia, such as the LipidSeq panel (11); this panel can be used to account for different variant types disrupting the main genes involved in the TG metabolic pathway, as well as non-coding polygenic contributors.

## Defining Hypertriglyceridemia

**Figure 1:**
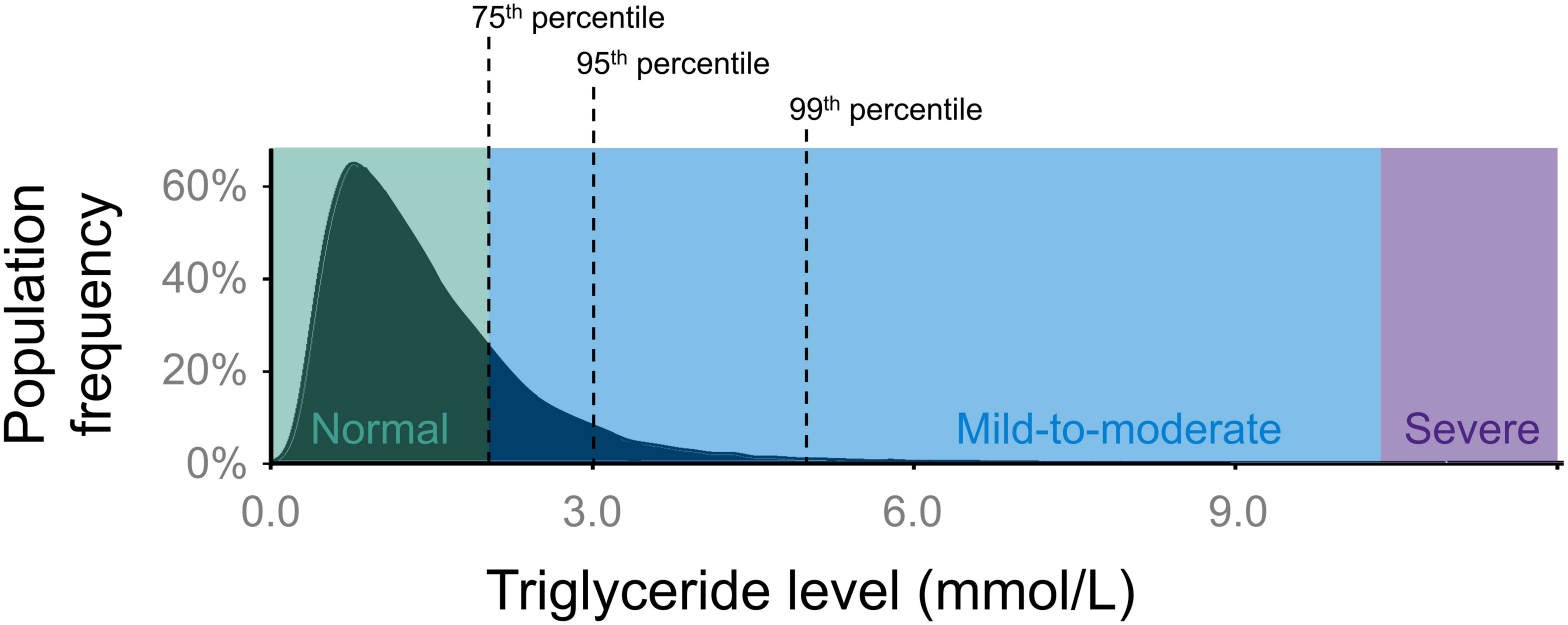
Population distribution of plasma TG levels. The distribution of TG levels has a positive (right) skew in the general population. In this review, normal TG levels are considered as <2.0 mmol/L (175 mg/dL), while anything above normal can be classified as hypertriglyceridemia. “Mild-to-moderate” hypertriglyceridemia is defined as 2.0–9.9 mmol/L (175–885 mg/dL), while “severe” hypertriglyceridemia is defined as ≥10.0 mmol/L (≥885 mg/dL). Severe hypertriglyceridemia can be further defined as FCS or MCM, depending on its genetic basis. The black dashed lines indicate the 75th percentile (2.0 mmol/L), 95th percentile (3.0 mmol/L), and 99th percentile (5.0 mmol/L) for TG levels. FCS, familial chylomicronemia syndrome; MCM, multifactorial chylomicronemia; TG, triglyceride.

Fasting TG levels follow a positive-skewed or right-skewed distribution in the general population (Figure 1) (12). A Canadian population survey showed that the 95th percentile for fasting TG levels is ~3 mmol/L and that ~1 person in 600 has fasting TG >10 mmol/L (>885 mg/dL) (13). These thresholds will vary between geographic areas and jurisdictions. A clinical diagnosis of “hypertriglyceridemia” is usually made by applying threshold values to the distribution of plasma TG levels (14). Different consensus committees recommend various threshold values for such discrete classifications as “mild,” “moderate,” and “severe” hypertriglyceridemia (14). For this review, we define “mild-to-moderate” and “severe” hypertriglyceridemia as 2.0–9.9 mmol/L (175–885 mg/dL) and ≥10.0 mmol/L (≥885 mg/dL), respectively (14), since these are the ranges used in recent cohort studies.

**Figure 2:**
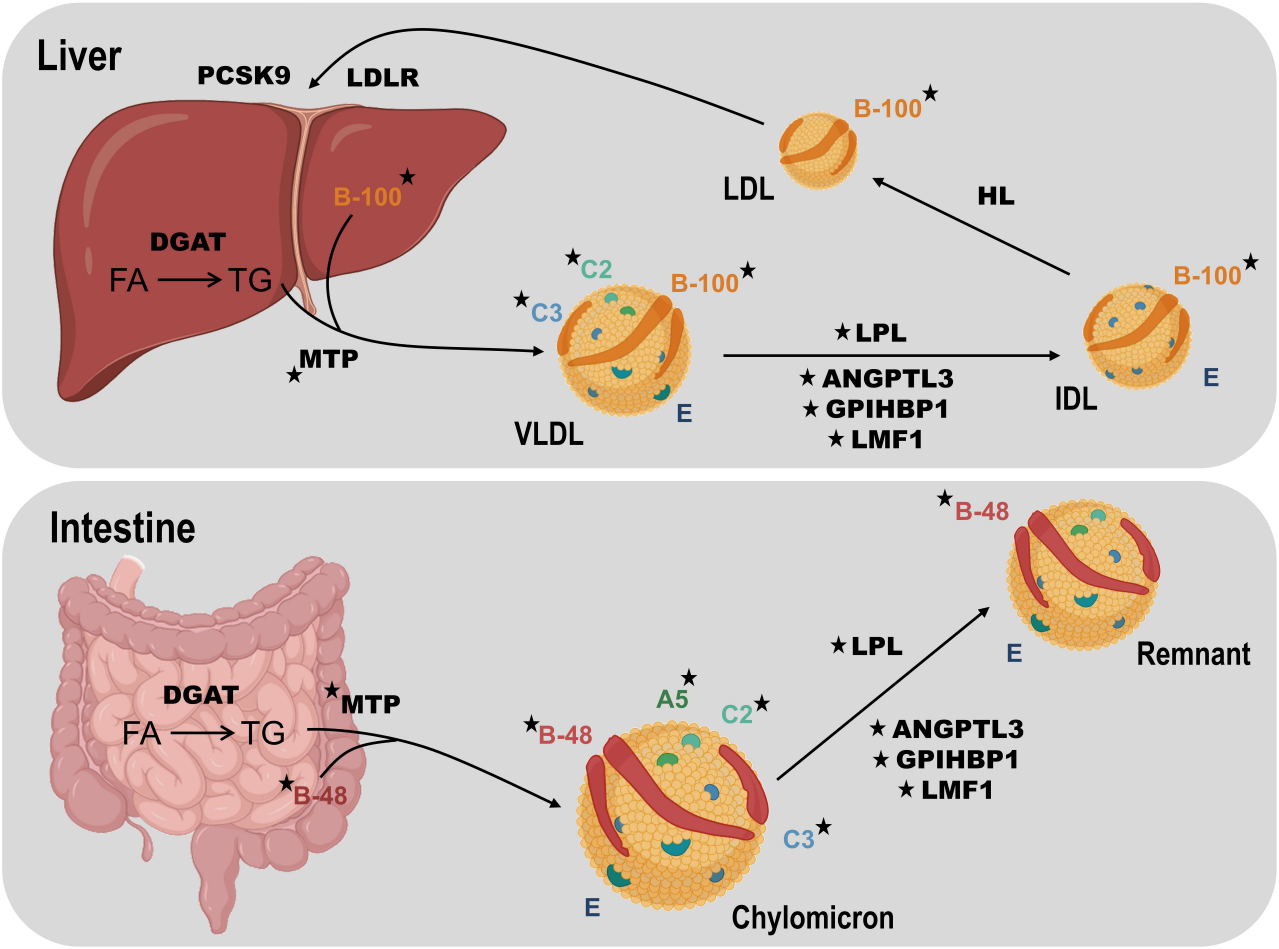
Overview of TG metabolism focusing on human disease genes. TG-rich lipoprotein assembly requires TG synthesis from fatty acids (FA) in the intestine or liver using tissue-specific isoforms of diacylglycerol acyltransferase (DGAT). Microsomal triglyceride transfer protein (MTP) fuses TG, cholesterol and phospholipids, with tissue-specific isoforms of apolipoprotein (apo) B: intestinal B-48 to form chylomicrons and hepatic B-100 to form very-low density lipoprotein (VLDL). Chylomicrons enter plasma through the thoracic duct while the liver secretes VLDL directly into the bloodstream. Lipoprotein lipase (LPL) is the key enzyme for hydrolysis of both circulating chylomicrons and VLDL, producing chylomicron remnants and intermediate-density lipoprotein (IDL) particles, respectively. Chylomicron remnant and IDL clearance by the liver is mediated by apo E (not shown). IDL is further hydrolyzed by hepatic lipase (HL) to generate low-density lipoprotein (LDL), which is cleared by the LDL receptor (LDLR), regulated in part by proprotein convertase subtilisin kexin 9 (PCSK9). Lipase maturation factor 1 (LMF1) chaperones LPL prior to secretion from adipocytes or myocytes. Glycosylphosphatidylinositol-anchored high-density lipoprotein binding protein-1 (GPIHBP1) translocates LPL across capillary endothelium and fastens it there. Apo C-II (C2) activates LPL, while apo A-V (A5) is a stabilizing cofactor. Apo C-III (C3) and angiopoietin like protein 3 (ANGPTL3) both inhibit lipolysis. Rare loss-of-function variants affecting genes encoding LPL, apo A-V, apo C-II, LMF1, and GPIHBP1 can cause familial chylomicronemia syndrome, while such variants in genes encoding apo B-100, MTP, apo C-III, and ANGPTL3 can result in low TG levels. Stars indicate gene products associated with altered TG levels. Elements of this figure were created using icons from the online source, BioRender. DGAT, diacylglycerol acyltransferase; FA, fatty acid; HL, hepatic lipase; IDL, intermediate-density lipoprotein; LDL, low-density lipoprotein; MTP, microsomal triglyceride transfer protein; VLDL, very-low-density lipoprotein.

To a first level of approximation, mild-to-moderate hypertriglyceridemia primarily reflects accumulation of very-low-density lipoproteins (VLDL) plus any related remnant particles, while severe hypertriglyceridemia usually indicates the presence of fasting chylomicrons in addition to excess VLDL plus any related remnants (Figure 2) (14). For this review, we consider that patients with plasma TG ≥10 mmol/L have chylomicronemia, and that the majority of these have “multifactorial chylomicronemia” (MCM) (15). A very small subset of severely hypertriglyceridemic patients has autosomal recessive chylomicronemia, i.e., FCS (16). The primary lipoprotein disturbance in FCS is accumulation of chylomicrons (17), while VLDL, remnants, and other lipoprotein species are scarce, due to the severe lipolytic blockade that compromises conversion of large TG-carrying particles into smaller lipoprotein species (18). One consequence is that plasma levels of apo B-100—the defining protein of both VLDL and low-density lipoprotein (LDL)—are depressed in FCS (19), reflecting paucity of downstream lipoprotein species in FCS. Conversely, in MCM, in which the blockade of lipolysis is only partial at most, VLDL and remnants are present in abundance, and plasma levels of apo B-100 are relatively high (20). For both FCS and MCM, plasma levels of apo B-48 are consistently elevated, reflecting this truncated form of apo B as the main protein constituent of chylomicrons.

Because chylomicrons are associated with an increased risk of pancreatitis, a TG level ≥10 mmol/L (≥885 mg/dL) is often used as a clinical rule of thumb to flag such risk (21). Pancreatitis risk rises even more steeply once TG levels exceed 20 mmol/L (22). In addition, risk of pancreatitis is somewhat increased even among patients with mild-to-moderate hypertriglyceridemia (23). However, in these patients a more pressing concern is increased risk of atherosclerotic cardiovascular disease (ASCVD) (24) and stroke (25). Similarly, patients with severe hypertriglyceridemia have increased risk of mortality from ASCVD (26), although it is axiomatic that the small subgroup of patients with FCS have reduced risk of ASCVD (15). The classical explanation is that: (1) physically large chylomicron particles cannot easily penetrate the endothelial barrier and cause atherosclerotic plaques; (2) other atherogenic lipoprotein species containing apo B-100 are in short supply (14, 27).

## Familial Chylomicronemia Syndrome

### Prevalence and Clinical Features

FCS is the only true monogenic form of hypertriglyceridemia. This was previously known as Fredrickson hyperlipoproteinemia type 1 or lipoprotein lipase (LPL) deficiency, although for decades, apo C-II deficiency was also recognized as a cause (Table 1) (28). The overall prevalence of FCS is reported to be quite rare, affecting ~1 in 100,000–1,000,000 individuals (29, 30). Recently, in a cohort of 563 patients with TG ≥10 mmol/L (≥885 mg/dL), we reported that 1.1% had rare biallelic variants indicating FCS (16). Furthermore, our clinical experience integrating DNA sequencing data with clinical assessments (11) confirms that FCS patients are exceedingly rare. Specifically, in our lipid clinic, we have molecularly identified a similar number of patients with FCS and homozygous FH (HoFH) (9). Since the population prevalence of HoFH is ~1 in 200,000–300,000 (31, 32), we would again estimate a roughly equal prevalence for FCS.

**Table 1:** Defining the molecular subsets of monogenic chylomicronemia.

**Form of monogenic chylomicronemia^a^**	**Inheritance pattern**	**Gene**	**Chromosomal coordinates^b^**	**OMIM number**
LPL deficiency	Autosomal recessive	*LPL*	Chr8: 19,759,228–19,824,770	609708, 238600
Apo C-II deficiency	Autosomal recessive	*APOC2*	Chr19: 45,449,239–45,452,822	207750, 608083
Apo A-V deficiency	Autosomal recessive	*APOA5*	Chr11: 116,660,083–116,663,136	145750, 144650, 606368
LMF1 deficiency	Autosomal recessive	*LMF1*	Chr16: 903,634–1,031,318	246650, 611761
GPIHBP1 deficiency	Autosomal recessive	*GPIHBP1*	Chr8: 144,295,068–144,299,044	612757
Infantile hypertriglyceridemia, transient	Autosomal recessive	*GPD1*	Chr12: 50,497,602–50,505,102	614480, 138420

a *Formerly described as type 1 HLP, also called familial chylomicronemia syndrome*.

b *Reported using GRCh37/hg19 coordinates. Apo, apolipoprotein; chr, chromosome; GPIHBP1, glycosylphosphatidylinositol-anchored high density lipoprotein-binding protein 1; HLP, hyperlipoproteinemia; LMF1, Lipase maturation factor 1; LPL, lipoprotein lipase; OMIM, Online Mendelian Inheritance in Man*.

FCS patients present with TG levels ≥10 mmol/L (≥885 mg/dL), and often much higher, due mainly to the abnormal accumulation of chylomicrons, which can be detected if blood is centrifuged or left to stand overnight: plasma appears lipemic, milky, or opalescent. The profound inherited disturbance of chylomicron processing leads to clinical manifestations early in life, including nausea, vomiting, failure to thrive and abdominal pain in infancy and childhood (30). Other clinical features of chylomicronemia appear variably in the life course and include lipemia retinalis, abdominal pain, nausea, vomiting, hepatosplenomegaly, and eruptive xanthomas on the trunk, extremities, and buttocks (33–35). The most serious health concern is an increased risk for life-threatening episodes of acute pancreatitis (30). Less common symptoms of FCS can also include diarrhea, intestinal bleeding, anemia, and even neurological features such as irritability and seizures (33, 34).

### Genetic Determinants

With DNA sequencing, we now know that in addition to the *LPL* and *APOC2* genes, biallelic LOF variants in *APOA5, LMF1*, and *GPIHBP1* genes can also cause this autosomal recessive disorder (36) (Table 1). Products of all five genes act in the catabolic pathway for chylomicrons (Figure 2), indicating how this critical metabolic chokepoint is sensitive to germline LOF variants. *LPL* encodes LPL, which has a focal non-redundant role in intravascular hydrolysis of TG-rich lipoproteins (18). *APOC2* and *APOA5* encode apo C-II and A-V, respectively (37, 38); rare LOF variants in either gene can lead to decreased interaction between LPL and TG-rich lipoproteins, particularly chylomicrons (18). Apo C-II is a co-factor that directly binds to LPL to activate hydrolysis, while apo A-V acts in a modulatory fashion to further enhance this activity (37). *LMF1* encodes lipase maturation factor 1 (LMF1), which traverses the endoplasmic reticulum and assists with the correct folding and maturation of LPL (39). *GPIHBP1* encoding glycosylphosphatidylinositol-anchored high density lipoprotein-binding protein 1 (GPIHBP1) transports and anchors secreted LPL to the endothelial surface, where LPL can become fully active after binding to apo C-II (40).

In FCS there is no reported causative gene that affects synthesis or production of TG-rich lipoproteins. Biallelic LOF variants in the five canonical genes for FCS lead to impaired hydrolysis of TG-rich lipoproteins, with subsequent increases in chylomicron particle numbers and markedly increased TG concentrations, since chylomicrons can carry such great quantities of the lipid. Among FCS patients, there have been >200 rare biallelic variants reported in the *LPL* gene, with ≤ 10 variants reported in each of the other genes (29).

### Genotype-Phenotype Correlation

Molecular and clinical attributes have been reported from one of the largest cohorts of FCS patients studied at the DNA level (36). These 52 patients came from a total of 13 different specialty clinics worldwide, emphasizing how extremely rare FCS actually is, given the logistical challenge to identify and recruit these patients. In these FCS patients, ~80% carried rare biallelic variants in *LPL* (36). Of the other 20%, almost half had rare biallelic variants in *GPIHBP1*, while the remainder had biallelic variants in the other three genes, or double heterozygosity for an *LPL* variant, plus a variant in another gene. More than 80% of causative variants identified in these patients were missense. About 5% were CNVs, of which deletions involving large regions of the *GPIHBP1* gene were most commonly seen (36). All FCS subjects had extremely high levels of TGs and chylomicrons, but very low levels of other lipoproteins, irrespective of the precise genotype. Clinical and biochemical features were largely similar across the different genetic etiologies. More than 80% of patients had a history of at least one episode of pancreatitis. Compared to those who had *LPL* variants, individuals without *LPL* variants had significantly higher postheparin LPL activity, higher 4-h postprandial insulin and C-peptide levels; and higher LDL cholesterol levels determined by ultracentrifugation.

## Multifactorial Chylomicronemia

### Prevalence and Clinical Features

In contrast to FCS, MCM is much more common, complicated and nuanced (15). Based on the reported Canadian prevalence of adults with severe hypertriglyceridemia (i.e., TG ≥ 10 mmol/L) (12, 13) together with our generalization that these individuals largely overlap with MCM patients, we would estimate a prevalence of MCM of ~1 in 600–1,000. MCM encompasses a much broader population of elevated TG-rich lipoprotein and remnant species than FCS. The clinical features in MCM include those related to chylomicronemia, such as lipemia retinalis, hepatosplenomegaly, eruptive xanthomas, nausea, vomiting, and abdominal pain (15). Pancreatitis occurs less commonly in MCM than in FCS; some estimates are ~10–20% over a lifetime (15), while rates in FCS have been estimated at ~60–80% (17). Also, because adults primarily express MCM, such pediatric features as failure to thrive are not typical.

### Genetic Determinants

MCM clearly has a genetic basis, but unlike FCS, in which recessive or biallelic variants in the five canonical genes are causative, the genetic factors in MCM are not deterministic, i.e., their presence does not guarantee the phenotypic expression of the trait. Rather, they are probabilistic in that they increase the risk of developing the condition, but do not guarantee its clinical expression. To illustrate this, a small proportion of normolipidemic people have identical genotypes of predisposing common and rare variant to patients with severe hypertriglyceridemia (14). Furthermore, many patients with MCM do not have any identified genetic factor, which may suggest that other non-canonical genes harbor influential variants that are being overlooked. Also, some other complexity could be involved, such as non-genomic effects (epigenetics, methylation), gene-gene, or gene-environment interactions.

There are two main types of genetic factors that increase the odds that a patient will develop MCM. The first are heterozygous rare large-effect variants in one of the five canonical genes involved in TG metabolism. The second is a high burden of common small-effect TG-raising SNP alleles from genome-wide association study (GWAS) loci; cumulatively, these common SNP alleles create a state of susceptibility for developing hypertriglyceridemia. We were able to concurrently evaluate both rare heterozygous variants and common SNPs (using a polygenic score) in hypertriglyceridemic cohorts by virtue of the design of our LipidSeq panel.

#### Rare Heterozygous Variants

In the pre-genomic era, John Brunzell carefully studied the parents of six children with biochemically proven complete LPL deficiency, i.e., children with FCS (41). Although genotyping was not performed, the phenotype indicated that these children were homozygous for *LPL* variants. This meant that the parents were obligate heterozygotes for *LPL* variants. In a total of 13 obligate heterozygotes, Brunzell found considerable phenotypic variation: (1) more than half of heterozygotes had normal TG levels, a few had mild hypertriglyceridemia, while two heterozygotes each had a TG reading >7 mmol/L on at least one occasion; and (2) LPL activity was widely variable, and in one individual was actually higher than in normal controls. Brunzell concluded that the heterozygous state for LPL deficiency was associated with variable expression of hyperlipidemia, with many phenotypically normal carriers. In family studies from Quebec, Pierre Julien et al. later found that among 43 DNA-proven heterozygotes for *LPL* variants, TG levels ranged from normal to severe, and that obesity and high fasting plasma insulin levels were associated with higher TG levels (42).

We have had a long interest in the association between rare heterozygous variants and hypertriglyceridemia. In 2008, we showed that ~10% of 110 patients with severe hypertriglyceridemia were heterozygotes for LOF variants in either *LPL* or *APOC2* (43). In 2010, we showed a 2-fold enrichment of rare heterozygous variants in four candidate genes, including *LPL* and *APOA5* in patients with hypertriglyceridemia (44). In 2012, we found an excessive prevalence of rare variants in *LPL, APOC2, GPIHBP1, APOA5*, and *LMF1* in patients with severe hypertriglyceridemia (45), again indicating that heterozygosity was a predisposing factor for, although not an absolute cause of severe hypertriglyceridemia or MCM.

Most recently, in 563 patients with severe hypertriglyceridemia whom we analyzed with LipidSeq, we found that 14.4% had heterozygous rare variants in *LPL, APOC2, GPIHBP1, APOA5*, and *LMF1* genes, compared to only 3.8% of normolipidemic controls, giving an odds ratio of 4.41 (95% confidence interval [CI]) 2.67–7.29, *P* < 0.0001) (16). We found 0.2% of individuals with severe hypertriglyceridemia had large-scale CNVs involving large portions of the *LPL* gene (46). Since participants were all unrelated adult individuals, we could not study phenotype-genotype co-segregation across generations in kindreds. But the fact that 3.8% of normal controls also had such heterozygous rare variants suggested that the variants were only partially or incompletely penetrant. The phenotype in heterozygotes for these rare variants was variable, ranging from normal to severe hypertriglyceridemia. Although there was a 4-fold increased risk in rare variant carriers having severe hypertriglyceridemia, this risk was not absolute in an individual patient. This echoes the phenotypic heterogeneity Brunzell and Julien observed in heterozygotes for LPL deficiency.

The totality of these data is consistent with the idea that in MCM, heterozygous rare variants in *LPL* and related genes are variably penetrant, despite being strongly associated to the phenotype. This is distinct from the situation with FH, in which rare heterozygous variants in *LDLR* and related causative genes are found to be strongly associated with the phenotype (47) and show consistently high penetrance.

For completeness, we mention that signals for enrichment of heterozygous rare variants in non-canonical (i.e., non-FCS) genes, such as *CREB3L3* (48, 49) and *GCKR* (50) have been detected in hypertriglyceridemic cohorts. While statistical association with higher mean TG levels is strong, these variants do not co-segregate with the lipid profile across generations in family pedigrees (48, 50), similar to the situation for heterozygous variants of *LPL*. The extent of contribution to severe hypertriglyceridemia by rare variants of these non-canonical genes has not been determined.

#### Common, Small-Effect Variants

The second type of genetic contributor to MCM is an increased burden of TG-associated SNPs (51). A few of these SNPs were identified early on in single candidate gene association studies (52) of plasma lipids. More recently, the full spectrum of SNPs associated with TG levels has been identified through large replicable GWASs (53–55) and their accumulation in patients is now quantified by polygenic scores (56, 57). Although these SNPs are mostly non-coding, it is thought that they influence gene regulation and expression, and may have a more peripheral, indirect impact of the metabolism of TG-rich lipoproteins and other related pathways (58). Unfortunately, it is very difficult to determine the functional mechanism driving these SNP associations and have not been fully characterized functionally; this poses a huge challenge for the genomics field in general. Despite incomplete understanding of the functional basis between an excess burden of SNPs and TG levels, their prevalence in patients with severe hypertriglyceridemia has been repeatedly demonstrated with extremely high statistical significance (16).

## Assessing Polygenic Risk: Single Gene, Single SNP Association Studies

Polygenic risk for hypertriglyceridemia is now quantified using a polygenic score (57) that includes a much greater number of markers. Previous SNP association studies permitted a partial glimpse—literally only one variant at a time—for a genotype-phenotype relationship that we now understand is based on the simultaneous contributions of a large number of variants.

Beginning more than 25 years ago, we used multivariate regression analysis as a precursor to the polygenic score. We initially showed that common SNP variants of multiple candidate gene loci were determinants of plasma TG (59) and of predisposition to hypertriglyceridemia (60, 61). In particular, at the start of the GWAS era, we showed in 132 patients with severe hypertriglyceridemia and 351 controls that genotypes for common variants in *APOA5, APOE, GCKR, TRIB1*, and *MLXIPL* were significantly associated with severe hypertriglyceridemia in multivariate regression analyses and that these variants collectively explained about one-quarter of the variation in disease status (60).

### Polygenic Scores in Severe Hypertriglyceridemia

A number of polygenic risk scores focused on TG levels have been published (53, 56, 62–70). Most recently, in a cohort of 563 patients with severe hypertriglyceridemia studied with our LipidSeq panel, we found that 32.0% of patients had a high polygenic score of TG-raising alleles across 16 loci (Table 2), compared to only 9.5% of normolipidemic controls, giving an odds ratio of 4.45 (95% CI 3.15–6.30, *P* < 0.0001) (16). As with the heterozygous rare variants in MCM, this genotype-phenotype relationship is probabilistic and not deterministic. Because ~10% of normal controls also had a high score (by definition, the 90th percentile in the general population was chosen as the threshold for “high polygenic risk”), it indicates that a high polygenic score on its own cannot fully explain the trait. While there is a significantly increased risk of developing hypertriglyceridemia when the score is high, this is by no means certain or absolute. This is similar to the pattern of increased risk seen with heterozygous rare variants; a high polygenic score is strongly associated with the cohort of patients with severe hypertriglyceridemia, but it cannot be used to diagnose an individual patient.

**Table 2:** SNP loci considered in the recently published 16-SNP polygenic score used to characterize the genetic basis of hypertriglyceridemia phenotypes.

**Chr:position**	**rsID**	**Gene**	**TG-raising allele**	**Variant ontology**
1:63025942	rs2131925	*DOCK7, ANGPTL3*	T	Intronic, upstream intergenic
1:230295691	rs4846914	*GALNT2*	G	Intronic
4:88030261	rs442177	*KLHL8, AFF1*	T	Downstream intergenic, intronic
5:55861786	rs9686661	*MAP3K1, ANKRD55*	T	Upstream intergenic, upstream intergenic
7:72982874	rs17145738	*MLXIPL*	C	Downstream intergenic
8:18272881	rs1495741	*NAT2*	G	Downstream intergenic
8:19844222	rs12678919	*LPL*	A	Downstream intergenic
8:126490972	rs2954029	*TRIB1*	A	Downstream intergenic
10:65027610	rs10761731	*JMJD1C*	A	Intronic
11:61569830	rs174546	*FADS1, FADS2, FADS3*	T	3′UTR, intronic, downstream intergenic
11:116648917	rs964184	*APOA1, APOC3, APOA4, APOA5*	G	Downstream intergenic, upstream intergenic, downstream intergenic, downstream intergenic
15:42683787	rs2412710	*CAPN3*	A	Intronic
15:44245931	rs2929282	*FRMD5*	T	Intronic
16:56993324	rs3764261	*CETP*	C	Upstream intergenic
19:19407718	rs10401969	*SUGP1*	T	Intronic
20:44554015	rs6065906	*PLTP*	C	Upstream intergenic

*Locus coordinates follow the GRCh37/hg19 build. For loci with multiple nearby genes, the variant ontology in reference to each gene is included. Chr, chromosome; SNP, single-nucleotide polymorphism; TG, triglyceride; UTR, untranslated region*.

Additional studies are needed to quantify the interactive effects between heterozygous rare variants and excess SNP accumulation. From this, we might be able to better appreciate the polygenic underpinnings of MCM. To understand the phenotype even further, demographic features, environmental factors, and other health conditions must also be brought into the equation. Within a single individual, TG levels can be influenced by age, sex, smoking status, obesity, diet, diabetes, insulin resistance, certain medications, alcohol intake, pregnancy, and activity level (14); many of these factors can change across time. It is very likely, although not yet proven, that non-genetic factors can force the expression of hypertriglyceridemia in an individual who is predisposed because they either carry a rare LOF variant or have a high polygenic score, or both.

## Mild-to-Moderate Hypertriglyceridemia

Very recently we showed that the probabilistic genotype-phenotype relationships seen in MCM are also seen in mild-to-moderate hypertriglyceridemia, which to a first approximation we consider to coincide phenotypically with former Frederickson type 4 hyperlipoproteinemia. We found significant enrichment of both rare heterozygous variants and high polygenic scores in 134 patients with mild-to-moderate hypertriglyceridemia compared to normolipidemic controls (71). Furthermore, these frequencies were intermediate between normolipidemic controls and severe hypertriglyceridemia. Specifically, 9.0% of patients with mild-to-moderate hypertriglyceridemia carried heterozygous rare variants, giving an odds ratio of 2.38 (95% CI 1.13–4.99; *P* = 0.021) compared to controls. Also, 24.6% of patients with mild-to-moderate hypertriglyceridemia had a polygenic SNP score in the 90th percentile, giving an odds ratio of 3.26 (95% CI 2.02–5.26; *P* < 0.0001) compared to controls. The further increments in odds ratios for patients with severe hypertriglyceridemia over patients with mild-to-moderate hypertriglyceridemia were 1.86 (95% CI 0.98–3.51; *P* = 0.032) and 1.63 (95% CI 1.07–2.48; *P* = 0.013) for heterozygous rare variants and extreme polygenic score, respectively.

## Other Hypertriglyceridemia Phenotypes

Although it is beyond the scope of this review, we briefly note that similar complex patterns of polygenic influences were previously reported in other disorders that have hypertriglyceridemia as part of the phenotype. Specifically, our 2010 study of combined hyperlipidemia (former Fredrickson type 2B) and dysbetalipoproteinemia (former Fredrickson type 3) showed significant enrichment of both rare heterozygous variants by ~2 and ~2.6-fold, respectively, and of a high polygenic score by ~2-fold for each disorder (72). Although the LipidSeq targeted panel was not used for this early work, the pattern of results strongly indicates a very similar genetic architecture for these other dyslipidemias for which hypertriglyceridemia is a defining component. This work needs to be repeated using next-generation sequencing methods. However, the overall findings are consistent with the previously proposed model of a complex genetic basis for most of the former Frederickson phenotypes (61, 73), except for type 1 (i.e., FCS) and type 2A (i.e., FH), which are both clearly monogenic.

## An Integrated View of the Genetic Determinants of Hypertriglyceridemia

**Figure 3:**
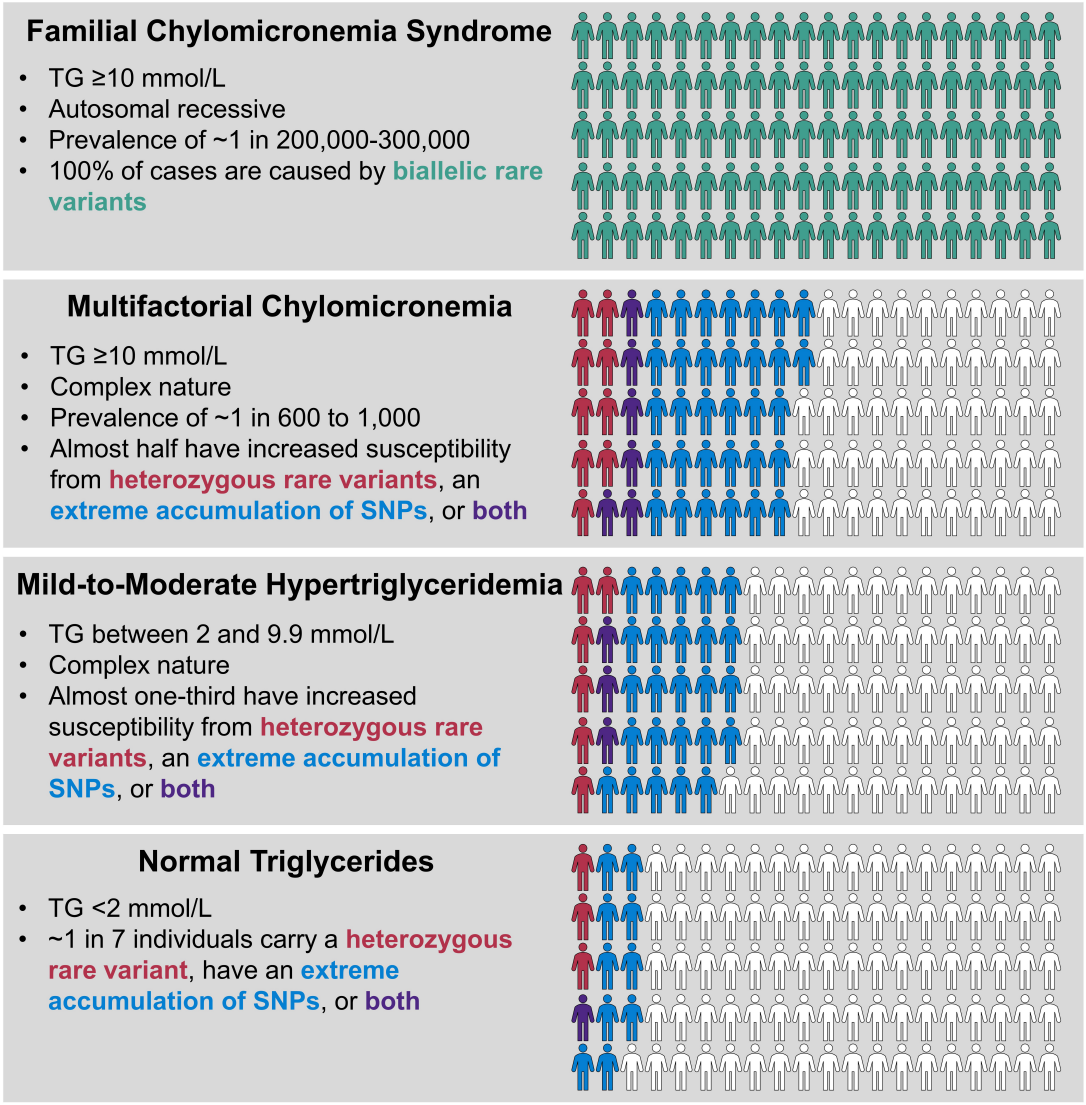
Genetic determinants of hypertriglyceridemia. FCS and MCM both present with severe hypertriglyceridemia with TG levels ≥10 mmol/l (≥885 mmol/L), while TG levels in mild-to-moderate hypertriglyceridemia are lower, ranging from 2.0 to 9.9 mmol/L (175–885 mg/dL). FCS is a very rare condition and is the only true classical monogenic phenotype, showing recessive inheritance with biallelic variants in *LPL, APOA5, APOC2, GPIHBP1*, and *LMF1* genes. MCM and mild-to-moderate hypertriglyceridemia have complex underlying genetics, showing statistical excess of heterozygous rare variants in *LPL, APOA5, APOC2, GPIHBP1*, and *LMF1* genes or extreme polygenic scores or both, compared to the normal population, with frequencies denoted by color scheme as shown. Each phenotype shows 100 individuals and the proportion of relevant genetic factors, but note the extreme differences in population prevalence for each condition, which is not depicted here (please also refer to Figure 1). Non-genetic, environmental factors also play a role in expression of the complex phenotypes. FCS, familial chylomicronemia syndrome; MCM, multifactorial chylomicronemia; TG, triglyceride.

Our working model for genetic determinants of hypertriglyceridemia is shown in Figure 3. FCS has a prevalence of ~1 in 200,000–300,000 and constitutes a very rare subgroup of patients with severe hypertriglyceridemia. FCS is relatively simple phenotypically, with elevations mainly in chylomicrons, but not other TG-rich lipoprotein fractions or remnants. It is a classical monogenic disorder that follows autosomal recessive inheritance and results from rare biallelic variants in one of five canonical genes, namely *LPL, APOC2, APOA5, LMF1*, and *GPIHBP1*. In contrast, MCM is at least 50–100-times as common as FCS and represents the large majority of molecularly defined patients with severe hypertriglyceridemia. MCM is complex phenotypically, with elevations in multiple species of TG-rich lipoprotein fractions and remnants. MCM is also complex genetically, with significantly increased proportions of individuals who carry rare heterozygous variants in any of the five canonical genes that underlie FCS and those who carry an extremely high burden of common small-effect TG-raising SNP alleles, integrated as polygenic scores. The heterozygous variants underlying hypertriglyceridemia would be considered as “variably penetrant” or “incompletely penetrant,” since a small proportion of normolipidemic controls also carries these variants, without clinical consequences. Similarly, 10% of normal controls have a high polygenic score, but have no clinical consequences.

The pool of patients with mild-to-moderate hypertriglyceridemia also has significant enrichment of both heterozygous rare variants and high polygenic scores, but at levels that are intermediate between normolipidemic controls and those with severe hypertriglyceridemia. This was reflected in the stepwise percentage increases in total genetic burden of 13.5, 33.6, and 47.4% in controls, mild-to-moderate hypertriglyceridemia patients and severe hypertriglyceridemia patients, respectively (71). In the remaining genetically undefined patients with each phenotype, possible underlying determinants include: non-mendelian genetic factors, such as epigenetics or mitochondrial genetics, higher order gene-gene interactions or gene-environment interactions, or the possibility that genes with previously unreported links to TG levels still exist that could contribute. The proportion of genetically undefined patients might also be reduced by evaluating additional non-canonical genes associated with TG or genes that underlie other medical conditions, such as diabetes, in which elevated TG are found secondarily. Finally, with the development of genome-wide polygenic or omnigenic scores, it may be possible to account for a greater proportion of susceptibility to hypertriglyceridemia than has been currently unaccounted for with the use of smaller polygenic scores (Table 2).

## Conclusion

Thus, compared to genetic dyslipidemias, particularly FH, hypertriglyceridemia is much more complex at the genetic level. Indeed FH is a poor model for conceptualizing the complex genetics of hypertriglyceridemia. Unlike FH, the presence of a heterozygous LOF variant is not diagnostic for FCS. Heterozygotes for rare large-effect variants have phenotypes ranging from normal (3.8% of normolipidemic controls) to mild-to-moderate hypertriglyceridemia (9.0% of such patients) to severe hypertriglyceridemia (14.4% of such patients). Furthermore, there is no such thing as autosomal dominant hypertriglyceridemia: within the same family, carriers of heterozygous *LPL* variants have biochemical phenotypes ranging from completely normal to severe hypertriglyceridemia. The technical term for this is “variable penetrance” or “incomplete penetrance,” in contrast to virtually “complete penetrance” that characterizes most *LDLR* variants in patients with FH. The frequency of heterozygous rare variants is a significant feature of the cohort of patients with hypertriglyceridemia but is not deterministic in an individual patient. In addition, the majority of patients ascertained with the same phenotype clinically, do not have these heterozygous variants. Finally, while a polygenic etiology in some cases of hypercholesterolemia has been clarified, this genetic mechanism is much more prevalent in severe hypertriglyceridemia. We observed a stepwise increase in frequency of those with an extreme polygenic SNP score of 9.5, 24.6, and 32.0% in controls, mild-to-moderate and severely hypertriglyceridemic patients, respectively.

Finally, monogenic chylomicronemia or FCS represents a very small but important subgroup of patients with severe hypertriglyceridemia. FCS is: (1) very rare in the population, with revised estimated prevalence of ~1 in 200,000–300,000; (2) quite rare among patients with severe hypertriglyceridemia, making up perhaps 1% of this cohort; (3) molecularly heterogeneous, caused by rare biallelic variants in five canonical genes, although biallelic *LPL* variants underlie the large majority of cases; (4) relatively homogeneous phenotypically across various molecular etiologies; and (5) differentiated from MCM by younger age of onset, absence of secondary factors, low apo B level and at least 5-fold greater risk of acute pancreatitis. FCS patients are likely to be candidates for new biologic therapies (30). However, the vast majority of severe hypertriglyceridemia cases with chylomicronemia that are not FCS have MCM, which is appropriately named given the more complex spectrum of causative genetic factors, and wide range of secondary non-genetic factors which can further influence the severity of the clinical presentation.

## Author Contributions

JD and RH contributed to the planning and writing of the manuscript. Both authors contributed to the article and approved the submitted version.

## Conflict of Interest

RH reports consulting fees from Acasti, Akcea/Ionis, Amgen, HLS Therapeutics, and Sanofi all unrelated to the topic of this manuscript. The remaining author declares that the research was conducted in the absence of any commercial or financial relationships that could be construed as a potential conflict of interest.
